# Some Aspects of Intestinal Obstruction

**Published:** 1908-06-13

**Authors:** 


### Points in Surgery.

### SOME ASPECTS OF INTESTINAL OBSTRUCTION.

### FÆCAL ACCUMULATION AND ITS TREATMENT.

Fæcal accumulation is a very important cause of intestinal obstruction. Accumulations of fæces are most commonly found in the rectum, the sigmoid, and the cæcum. The accumulation may appear as one or more isolated masses, or may fill the colon as a continuous column from the rectum upwards. Smaller masses may be retained in the sacculi, and these tend to become inspissated and hardened until in extreme cases they have the consistence of dry mortar.

#### Factors in Causation.

The two factors which appear to be most constantly responsible for the condition are a weakness of the bowel wall and the state of the bowel contents. The weakness of the wall may be congenital, but is more often acquired and is associated with weak abdominal muscles and the prolonged use of aperients. The bowel contents are modified by chronic dyspepsia, often the result of the bolting of food, especially at irregular hours, and the consumption of masses of indigestible matter, both of which predispose to constipation.

The condition is much more common in females than in males, and is most often found in hysterical patients who have passed middle life. It is usual to find a history of chronic dyspepsia, imperfect teeth, and long-continued neglect of the bowels. The patients are liable to habitual constipation, and the bowels are seldom opened without purges. As a general rule, enormous quantities of fæcal material are passed by these means at intervals of many days, and not infrequently there is a spurious diarrhœa after the bowels are open, owing to the catarrhal state of the intestine above the mass. This may go on for years without causing any symptom severer than malaise.

#### Clinical Course.

In other cases the evacuations become less and less frequent, and the patient complains of a sense of fullness and weight in his abdomen, accompanied by anorexia, fœtor of the breath, and a foul tongue. He is troubled by indigestion, flatulence, and eructations, and has a bad taste in his mouth. In cases of prolonged constipation the phenomena of auto-intoxication become apparent. The skin looks dull, greasy, and unwholesome, and has an unpleasant odour. It is not unlike the skin seen in chlorosis. The conjunctivæ are often yellow, owing to the absorption from the gut of chromogens, which are a product of decomposition. Other symptoms complained of are headache, malaise, and vomiting. There is often a definite pyrexia, which is noticed after the fæcal masses have been disturbed by an evacuation.

If great distension of the abdomen supervenes there may be dyspnœa and palpitations, and in bad cases this becomes progressively worse until distended coils are seen through the parietes. True peristalsis is rarely visible, and then only in advanced cases: it is never so marked as in organic stricture. Paroxysms of colic add to the patient's distress, and he suffers from nausea and vomiting, which may even become stercoraceous. If the constipation is not relieved he dies eventually of the symptoms of an unyielding obstruction, unless he is previously carried off by intestinal septicæmia.

It must be remembered, however, that absolute constipation may persist for a perfectly amazing length of time without causing a fatal result. In fact, some of the records are hardly credible. One case is reported (from America) of a man aged twenty-six, always liable to habitual constipation, who had no evacuation of the bowels for eight months and sixteen days and yet survived! And Holmes refers in his "System of Surgery" to the case of a woman, aged thirty-five, who habitually had only one evacuation in every three months.

In all cases of fæcal accumulation there is one important diagnostic feature, and that is the tumour formed by the masses of retained fæces. On palpation it is hard, uneven, globular in shape, and, as a rule, not tender. When situated in a movable part of the intestine the tumour is itself mobile. When the patient is examined under an anæsthetic it can be felt to be of a doughy consistency.

#### Treatment.

In the treatment of fæcal impaction the most reliable and certainly the safest means of getting rid of the obstruction is the use of enemata. Soap and water answers the purpose as well as any other ingredient. It should be given through an ordinary tube inserted some four or five inches into the rectum. Some authorities disapprove of the use of the long rectal tube, as they think it likely to be a dangerous instrument in unskilful hands. The patient should lie on his back, with his buttocks raised, and large quantities of fluid should be introduced through a funnel. Once or twice a day an ordinary enema may be administered containing some intestinal irritant such as a drachm of turpentine or fifteen grains of asafœtida, to induce movements of the lower bowel. The author has obtained very satisfactory results from an enema composed as follows:—Magnesii sulphatis ℥iss. and glycerin ℥iss., made up to five ounces with warm water.

Aperients given by the mouth must be used with great caution, because, if their action is at all violent, they tend to produce colicky pains. Small doses of saline cathartics, which produce an exudation of fluid into the intestine, are valuable, and one of the most reliable is magnesium sulphate in drachm doses. When there is evidence that the fæcal masses are becoming softer and are shifting their position under this treatment, more drastic purges, such as calomel or castor oil, may be resorted to in order to facilitate the discharge of the material. Belladonna and strychnine may be given subsequently to increase the tone of the muscular coat of the intestine.

Gentle abdominal massage and faradism are useful accessory measures in the treatment of this condition. The best form of faradism to employ is what is known as the recto-abdominal. One pole is placed on the abdomen, and the other, a copper ball mounted on an insulating handle, is inserted into the rectum. Many surgeons think highly of this form of treatment. It must be remembered that the dislodgement of long-retained fæcal masses is attended by considerable constitutional disturbance due to septic absorption, and it is well to counteract this with ten-grain doses of salol three times a day.

Operative procedures for uncomplicated fæcal impaction are hardly ever called for; indeed, they are only justifiable when all other methods of treatment have failed, and the diagnosis has become so doubtful that it is necessary to ascertain whether there is an organic stricture or not.

